# Functional Characterization of Olfactory Receptors in the Thyroid Gland

**DOI:** 10.3389/fphys.2021.676907

**Published:** 2021-07-27

**Authors:** Daniel Weidinger, Nikolina Jovancevic, Denise Zwanziger, Sarah Theurer, Judith Hönes, Dagmar Führer, Hanns Hatt

**Affiliations:** ^1^Department of Cell Physiology, Ruhr-University Bochum, Bochum, Germany; ^2^Department of Endocrinology, Diabetes and Metabolism, University Hospital Essen, University of Duisburg-Essen, Essen, Germany; ^3^Institute of Pathology, University Hospital Essen, University of Duisburg-Essen, Essen, Germany

**Keywords:** olfactory receptor, thyroid cancer, G protein-coupled receptor, CRISPR/Cas9, calcium imaging

## Abstract

Olfactory receptors (ORs) are almost ubiquitously expressed in the human body. However, information about their functions in these tissues is lacking. To date, no functional characterization of expressed ORs in the human thyroid has been performed. In this study, we detected and compared the expression of OR2H2 and OR2W3 in healthy and malignant cell lines and their corresponding tissues, respectively. We demonstrated that stimulation of ORs by their specific ligand resulted in a transient increase in intracellular calcium and cAMP concentrations. In the case of OR2H2, the downstream signaling cascade analysis revealed that adenylate cyclase (AC) and phosphoinositide phospholipase C (PLC) were involved. Furthermore, OR2H2 and OR2W3 activation affected migration, proliferation, and invasion. These are the first insights that ORs influence physiology-relevant processes in the healthy and malignant thyroid.

## Introduction

The thyroid gland is responsible for the synthesis and secretion of the thyroid hormones (THs), which are essential for the development and normal organ function in the body. Thyroid differentiation, function, and proliferation are regulated by a plethora of endocrine and paracrine factors, including foremost thyroid-stimulating hormone (TSH), which activates TH synthesis and thyroid growth *via* binding to the G protein-coupled TSH-receptor.

Olfactory receptors (ORs) belong to the supergene family of G protein-coupled receptors (GPCR; Feldmesser et al., 2006; De La Cruz et al., 2009). Contrary to initial expectations, their expression is not limited to the nasal epithelium, as ORs could already be detected in various tissues such as the lung, heart, skin, and kidney (Giandomenico et al., 2013; Kalbe et al., 2016; Jovancevic et al., 2017a; Wojcik et al., 2018). In many tumor tissues, ORs are overexpressed and discussed as potential biomarkers (Maßberg and Hatt, 2018; Weber et al., 2018; Lee et al., 2019). The exact function of these extra-nasally expressed ORs has not yet been fully clarified, but their involvement in numerous cellular processes such as proliferation (Busse et al., 2014; Kim et al., 2015; Jovancevic et al., 2017b; Kalbe et al., 2017; Weber et al., 2017), apoptosis (Manteniotis et al., 2016a) or secretion processes (Braun et al., 2007) was demonstrated. One interesting example is the OR2H2 that was detected in human myoblasts. Activation by its specific ligand aldehyde 13-13 led to an inhibition of myoblast differentiation (Kalbe et al., 2018). Another potential deorphanized but not yet functionally characterized olfactory receptor is OR2W3, for which a strong expression has previously been found in the thyroid gland (Flegel et al., 2013).

To investigate a potential use and reveal the physiological function of OR2H2 and OR2W3 in the human thyroid gland, the exact signaling cascades of these are needed. To date, no characterization of an OR has been performed in the human thyroid gland. Since the effects of the same olfactory receptor differ in the tissues, no information on the signaling pathways or influenced physiological effects was known so far (Busse et al., 2014; Manteniotis et al., 2016a). This work shows for the first time the influence of ORs on cellular processes in thyroid cells and compares them with cells of follicular and papillary thyroid cancer (PTC and FTC).

## Materials and Methods

### Thyroid Samples

Thyroid tissue samples from 16 thyroid cancer patients were investigated. Histological classification of tissue specimen according to WHO criteria was obtained by certified pathologists (Institute of Pathology, University Hospital Essen). ORs mRNA expression was investigated in frozen tissues of eight follicular thyroid carcinomas (FTC), eight papillary thyroid carcinomas (PTC), and corresponding non-neoplastic tissue thyroid specimen.

### Cell Culture

For cell culture experiments, the following human cell lines were used: thyroid follicular epithelial cells Nthy-ori 3-1, follicular thyroid carcinoma cells, FTC 133, and papillary thyroid carcinoma cells, BCPAP. Nthy-ori 3-1 cells are derived from lobectomy (Lemoine et al., 1989), FTC 133 are derived from a metastasis near the primary tumor (Hoelting et al., 1994). BCPAP are derived from a poorly differentiated thyroid carcinoma (Fabien et al., 1994). All cell lines were purchased from the European Collection of Cell Cultures (ECACC, Salisbury, United Kingdom), re-authenticated by the mRNA expression profile of different thyroid hormone markers (*TG*, *TPO*, *NIS*, and *THOX1*) and negatively analyzed for mycoplasma contamination (AppliChem GmbH, Darmstadt, Germany). Cells were used between passages 3 and 7. Nthy-ori 3-1 were cultured in RPMI 1640 (Thermo Fisher Scientific, Waltham, United States), FTC 133 cells were cultured in Ham’s F12 (Thermo Fisher Scientific), and BCPAP cells were cultured in DMEM (Thermo Fisher Scientific), all supplemented with 10% fetal bovine serum (FBS, Invitrogen, Carlsbad, United States). The starvation medium contained 2% FBS. Cells were grown at 37°C and 5% CO_2_.

### RNA Isolation and Reverse Transcription PCR

RNA was extracted from frozen thyroid tissues (FTC, PTC, and healthy surrounding tissues) and cells (Nthy-ori 3-1, FTC 133, and BCPAP) using the RNeasy Mini Kit (Qiagen, Hilden, Germany) according to the manufacturer’s protocol. An additional DNase digestion using TURBO DNA-free™ Kit (ThermoFisher Scientific) was performed to remove genomic DNA contamination. The GoTaq® qPCR Master Mix (Promega, Madison, United States) was used to perform reverse transcription PCR. Oligonucleotides of OR2H2 and OR2W3 were designed using Primer Blast (NCBI) and synthesized by ThermoFisher Scientific. The primers used for RT-PCR were as follows: OR2H2: 5ꞌ-GGTCCCAGCTCTAATTCGACT-3ꞌ, 5ꞌ-CACTGCCCAGGTAATGGCTC-3ꞌ, OR2W3: 5ꞌ-CCTCCACACCCCCATGTACT-3ꞌ, 5ꞌ-CCAGAACCCAGGAACAGGAAG-3ꞌ; TBP: 5ꞌ-TATAATCCCAAGCGGTTTGC-3ꞌ, 5ꞌ-GCTGGAAAACCCAACTTCTG-3ꞌ. The following temperature cycle profile was used: 3 min at 95°C, followed by 40 cycles of 45 s at 95°C, 45 s at 60°C, and 45 s at 72°C and a final elongation of 10 min at 72°C. For normalization, the reference gene *TBP* (TATA-box binding protein) was used.

### Calcium Imaging

Thyroid cells were incubated for 30 min in loading buffer containing Ringer’s solution (140 mM NaCl, 5 mM KCl, 5 mM CaCl_2_, 5 mM MgCl_2_, and 1 mM HEPES) and Fura-2-AM (Invitrogen, Carlsbad, United States). Subsequently, the loading buffer got removed and the cells were rinsed with Ringer solution. The analysis was performed using an Olympus fluorescence microscope (IX71, Olympus, Shinjuku, Japan), a polychromator (MT 20, Olympus), and 10x objective (Olympus). The images were recorded at integrated fluorescence ratios (*f*_340_/*f*_380_) *via* the Cell-R Software (Olympus). The analyzed area was randomly selected. The inhibitors: SQ22536 (10 μM, Abcam, Cambridge, United Kingdom), U-73122 hydrate (2 μM, Sigma-Aldrich; St. Louis, United States), and L-cis Diltiazem (100 μM, Abcam) were dissolved in DMSO or aqua dest according to the manufacturer’s instructions.

### cAMP Assay

The cells were seeded into 96-well plates (ThermoFisher Scientific) and cultivated for 48 h. After cultivation, the cells were stimulated for 30 min with the odorants diluted to different concentrations (100, 250, 500, and 1,000 μM). Forskolin (10 μM, Sigma-Aldrich) was used as a positive control and the solvents served as the negative control. The cAMP levels were determined using the cAMP-Glo™ Kit (Promega) according to the manufacturer’s protocol and measured *via* a plate reader (Packard, PerkinElmer, Waltham, United States). The determined values were normalized to the control (0.1% ethanol/DMSO).

### CRISPR-Cas9

The knockout of the ORs was performed using the CRISPR-Cas9-method. To create inactive ORs, the “Crisp-Era” tool was used to find important structural sequences. The recognition sequences were (OR2H2-1.1: CCGGGCCCACTAGGGTTAGGAGGT and OR2H2-1.2 AAACACCTCCTAACCCTAGTGGGC). They include a green fluorescent protein (GFP) coding sequence and were inserted by the Guide-it™ CRISPR/Cas9 System (Green; Takara, St-Germain-en-Laye, France). Transfection was performed by using Lipofectamine™ 2000 (ThermoFisher Scientific) according to the manufacture’s protocol. Cells were analyzed *via* Calcium Imaging 48 h post-transfection. Successfully transfected cells (about 30%) could be recognized through the expression of the GFP (ZSGreen1) and were normalized to the untransfected cells afterward.

### Immunocytochemical Staining

For immunocytochemical staining, the following antibodies were used: rabbit anti-OR2H2 (ABIN6100219, Antikörper.de, Aachen, Germany) and rabbit anti-OR2W3 (PA5-61043, ThermoFisher Scientific). The antibody specificity has already been demonstrated using recombinant-expressed rho-tagged ORs in Hanna3A cells (Flegel et al., 2015). The thyroid cells were seeded on coverslips and maintained as previously described (Jovancevic et al., 2017a). The cells were fixed through 20-min incubation at 4°C in 4% paraformaldehyde. Afterward, the cells were washed and permeabilized with a solution containing PBS −/− and Triton X-100 (PBST). The cells were blocked using PBST + 1% gelatin from cold-water fish skin (Sigma-Aldrich) and 10% goat serum. The primary antibodies (1:100 diluted) were incubated overnight at 4°C in PBST + 1% gelatin and 2% goat serum. To allow detection, cells were incubated with fluorescent goat anti-rabbit antibodies (1:1,000, ThermoFisher Scientific), Alexa Fluor™ 488 Phalloidin (1:200, ThermoFisher Scientific) and Hoechst 33258 (1:1,000, ThermoFisher Scientific), for 45 min in PBST + 2% gelatin and 2% goat serum. Cells were rewashed with PBST and mounted on a slide with ProLong™ Gold Antifade Mountant (ThermoFisher Scientific). Images were captured by using an LSM510 Meta confocal microscope (Zeiss, Jena, Germany).

### Transwell Migration Assay

Nthy-ori 3-1 (10,000 cells/Insert), FTC 133 (5,000 cells/Insert), and BCPAP (10,000 cells/Insert) cells were seeded onto cell culture Inserts (Greiner Bio-One GmbH, Essen, Germany) into 24-well plates in 200 μl of the respective growth medium (upper compartment). The lower compartment contained 700 μl of the respective growth medium. Cells were incubated for 48 h. The medium of the compartment above was replaced by the respective starving medium containing nerol (250 μM) or aldehyde 13-13 (250 μM). Transwell migration was performed for 24 h at 37°C and 5% CO_2_. The medium was replaced, and a cotton bud removed non-migrated cells. Then cells were detached with 0.25% trypsin/EDTA (Invitrogen) stained by trypan blue and counted using a hemocytometer. As vehicle control, DMSO or ethanol was used.

### Transwell Invasion Assay

Cell culture inserts (Greiner Bio-One GmbH) were placed onto 24-well plates and coated with matrigel (200 μg/ml, BD Bioscience, Franklin Lakes, United States) for 2 h at 37°C. Nthy-ori 3-1 (10,000 cells/Insert), FTC 133 (5,000 cells/Insert) and BCPAP (10,000 cells/Insert) cells were seeded onto the matrigel-coated membrane in 200 μl of the respective starvation medium. The lower compartment contained 700 μl of the respective normal growth medium containing nerol (250 μM) or aldehyde 13-13 (250 μM). Transwell invasion was performed for 24 h at 37°C and 5% CO_2_. The same protocol as described in the transwell migration assay was used. The percentage of invaded cells/well normalized to the respective control was determined.

### Proliferation Assay

For proliferation assays, Nthy-ori 3-1 (10,000 cells/well), FTC 133 (5,000 cells/well), and BCPAP (10,000 cells/well) cells were seeded onto 96-well plates and were cultured in the respective normal growth media containing nerol (500 μM) or aldehyde 13-13 (500 μM) for 48 h at 37°C and 5% CO_2_. 5-bromo-2ꞌ-deoxyuridine (BrdU) labeling solution was prepared by following the manufacturer’s protocol (5-Bromo-2ꞌ-deoxy-uridine Labeling and Detection Kit III, Roche, Germany). BrdU incorporation for 3 h was measured by an ELISA reader (Molecular Devices, San Jose, CA, United States) at 405 nm and a reference wavelength of 490 nm. The percentage of proliferating cells normalized to the respective control was determined.

### Odorants

Aldehyde 13-13 was provided by Henkel (Düsseldorf, Germany), while nerol was received by Sigma Aldrich. The odorants were prediluted in ethanol (aldehyde 13-13) or DMSO (nerol), in which the final solvent concentration was ≤0.1%.

### Statistical Analysis

Cell culture experiments were performed in triplicates. GraphPad Prism 9 was used for statistical analysis of the received data. Results are shown as mean ± SEM. The Shapiro-Wilk normality test was used to determine whether a normal distribution was present. The significance levels were calculated with a two-tailed paired *t*-test (Gaussian distribution), Wilcoxon matched-pairs signed-rank test (nonparametric distribution), or Mann–Whitney U-test. The comparison between multiple groups was calculated by using one-way ANOVA (Gaussian distribution), Kruskal-Wallis, or Friedman test (nonparametric distribution), with additional *post hoc* Dunnett (Gaussian distribution) or “Two-stage step-up method of Benjamini, Krieger, and Yekutieli” (nonparametric distribution). The dose-response curve and the EC_50_ value were calculated using the 3-parameter Hill model. Differences were considered significant if ^*^p ≤ 0.05, ^**^p ≤ 0.01, and ^***^p ≤ 0.001.

## Results

### Expression Profile of OR2H2 and OR2W3 in Human Thyroid Tissue and Cell Lines

#### Olfactory Receptors 2H2 and 2W3 Are Expressed in Benign and Malignant Human Thyroid Tissue

**Figure 1:**
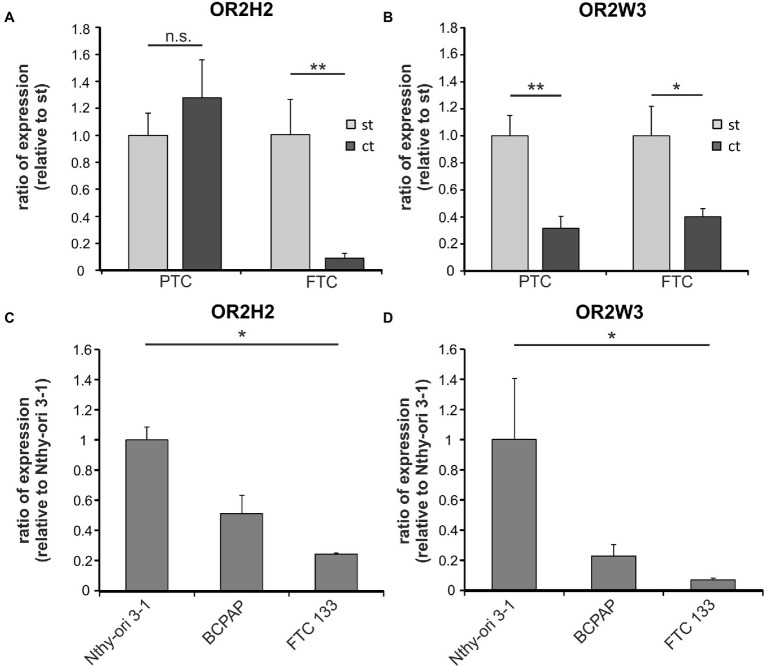
Expression of OR2H2 and OR2W3 in human thyroid tissues and cell lines. **(A,B)** Detection of *OR2H2* and *OR2W3* mRNA in human tissues of papillary thyroid carcinoma (PTC *n* = 8) and follicular thyroid carcinoma (FTC *n* = 8). Cancer tissue (ct) was compared to normal surrounding tissue (st) *via* quantitative RT-PCR. **(C,D)** mRNA expression level of *OR2H2* and *OR2W3* in Nthy-ori 3-1, BCPAP, and FTC 133 cell lines determined by quantitative RT-PCR (*n* = 3). The expression of *OR2H2* and *OR2W3* in FTC 133 and BCPAP was compared to Nthy-ori 3-1 cells. The mean values are shown with the corresponding SEM. Mann-Whitney-U-Test used to compare surrounding and malign tissues, while Kruskal-Wallis test with *post hoc* “Two-stage step-up method of Benjamini, Krieger, and Yekutieli” was used to compare expression levels between the cell lines: ^*^p ≤ 0.05 and ^**^p ≤ 0.01; n.s. = not significant.

To identify OR expression in the human thyroid gland, we first analyzed next-generation sequencing (NGS) data previously published by Flegel et al. (2013). The two receptors OR2H2 and OR2W3 were selected for further experiments according to their expression level and because activating ligands for both ORs are known (Flegel et al., 2015; Kalbe et al., 2018). To verify the ORs’ expression, human thyroid tissue samples of papillary (PTC) and follicular thyroid cancer (FTC), as well as the healthy surrounding tissue, were analyzed by RT-PCR. Both ORs were detectable in human thyroid tissues with significantly higher mRNA expression in healthy surrounding thyroid tissue than in carcinoma tissues (Figures 1A,B) with the exception that the expression of *OR2H2* mRNA in PTC remained unchanged.

#### OR2H2 and OR2W3 Expression in Human Thyroid Cell Lines

To investigate the functional relevance of *OR2H2* and *OR2W3* expression in benign and malignant human thyroid cell lines, Nthy-ori 3-1 cells derived from healthy human thyroid, BCPAP cells (PTC-derived), and FTC 133 cells (FTC-derived cells) were used. First, the expression and localization of both ORs were determined on mRNA and protein level. In all three cell lines, *OR2H2* and *OR2W3* were detected on mRNA level. Interestingly, *OR2H2* and *OR2W3* mRNA expression was significantly lower in FTC 133 cells comparing to Nthy-ori 3-1 cells (Figures 1C,D).

**Figure 2:**
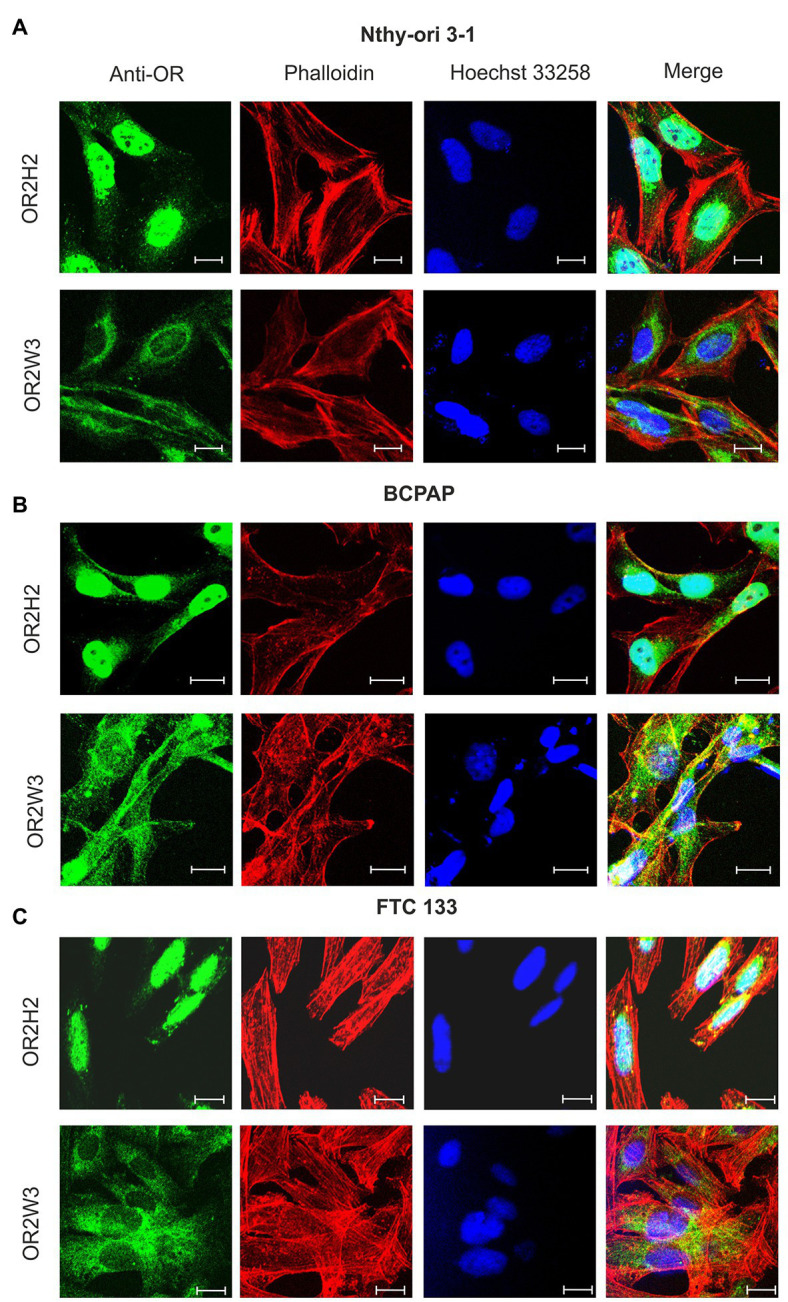
Localization of OR2H2 and OR2W3 in human thyroid cell lines. Nthy-ori 3-1 **(A)**, BCPAP **(B)**, and FTC 133 **(C)** cells were stained with specific antibodies against OR2H2 and OR2W3 (green), the cytoskeleton (red) was marked with phalloidin, whereas the cell nucleus (blue) was stained with Hoechst 33258. The scale bar corresponds to 20 μm.

Immunocytochemical staining confirmed the expression of the receptors OR2H2 and OR2W3 on protein level in these cell lines (Figure 2). Besides, a pronounced nuclear localization of OR2H2 was observed, whereas OR2W3 was predominantly located in the cytosol and plasma membrane.

Based on these findings, Nthy-ori 3-1, FTC 133, and BCPAP cells were selected as suitable *in vitro* models for further characterization of the functional impact of OR2H2 and OR2W3 in human benign and malignant thyroid cells.

### Aldehyde 13-13 Induces a Dose-Depending Increase of Intracellular Calcium Level in Thyroid Cells by OR2H2 Stimulation

**Figure 3:**
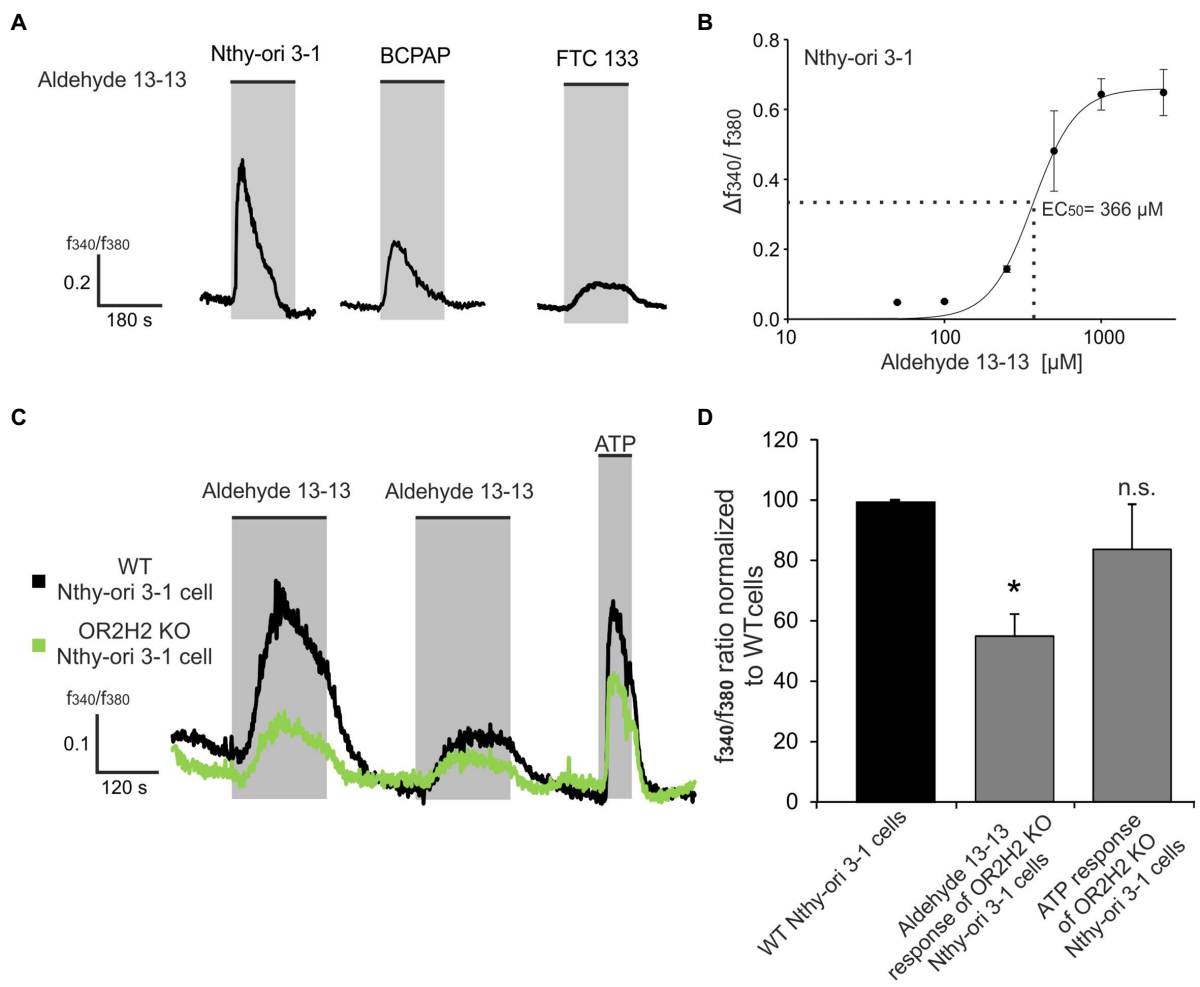
Aldehyde 13-13 mediates an increase of the intracellular Ca^2+^ concentration by the stimulation of OR2H2 in Nthy-ori 3-1 cells. **(A)** Exemplary traces of Ca^2+^ responses induced by stimulation with 500 μM aldehyde 13-13 in the thyroid cell lines. **(B)** The dose-response curve was prepared for the Nthy-ori 3-1 cell line. The calculated fluorescence ratio of *f*_340_/*f*_380_ (magnitude) was plotted against the aldehyde 13-13 concentration. The curve was fitted using Hill’s 3rd equation. The half-maximum Ca^2+^ increase occurred at a concentration of 366 μM aldehyde 13-13. **(C)** Exemplary Ca^2+^ imaging traces of Nthy-ori 3-1 cells transfected with CRISPR/Cas9 system for a knockout of the OR2H2 gene (KO, green) and wild type cells (WT, black). Cells were stimulated with aldehyde 13-13 (500 μM) and ATP (100 μM) as control. **(D)** The magnitudes of the Ca^2+^ response of transfected cells were normalized to the WT cells in the same measurement. The mean values are shown with the corresponding SEM. Data significance was calculated by comparing aldehyde 13-13 or ATP induced magnitudes of KO with WT Nthy-ori 3-1 cells using Wilcoxon matched-pairs signed-rank test. *n* = 6–9, ^*^p ≤ 0.05, n.s. = not significant.

Activation of an OR by its specific ligand leads in most cell types to an alteration of the intracellular Ca^2+^ level (Maßberg et al., 2015; Manteniotis et al., 2016b; Kalbe et al., 2017). Therefore, we used the Ca^2+^ imaging technique to analyze whether OR2H2 and OR2W3 were functionally expressed in the thyroid cell lines. For this purpose, the already known ligands aldehyde 13-13 for OR2H2 and nerol for OR2W3 were applied to the cells at a concentration of 500 μM (Figure 3; Supplementary Figure S1; Flegel et al., 2015; Kalbe et al., 2018). In the following Ca^2+^ imaging experiments, we focused on the result of aldehyde 13-13 induced signal in Nthy-ori 3-1 cells in more detail, since these were more suitable due to a more stable reaction, and thus, an investigation of all desired inhibitors was possible. Beyond the scope of this study, the observed effects in the other cell lines and with nerol have yet to be confirmed.

The stimulation with aldehyde 13-13 and nerol resulted in a transient increase of the intracellular Ca^2+^ concentration in each of the three thyroid cell lines (Figure 3A; Supplementary Figure S1). The aldehyde 13-13 induced increase was dose-dependent with an EC_50_ value of 366 μM in Nthy-ori 3-1 (Figure 3B). The application of the solvent ethanol with a concentration of 0.1% did not trigger any Ca^2+^ response (Supplementary Figure S2).

The CRISPR/Cas9 system enabled a knockout of OR2H2 in Nthy-ori 3-1 cells, clarifying its specific role in aldehyde 13-13 mediated effects. A GFP-plasmid as transfection control allows the identification of cells with a potential knockout of the *OR2H2* gene. A knockout of the OR2H2 resulted in a significantly reduced aldehyde 13-13 Ca^2+^ response compared to control, whereas the ATP response remained unaffected (Figures 3C,D).

### Aldehyde 13-13 Induced Signal Pathway in Nthy-ori 3-1 Cells

**Figure 4:**
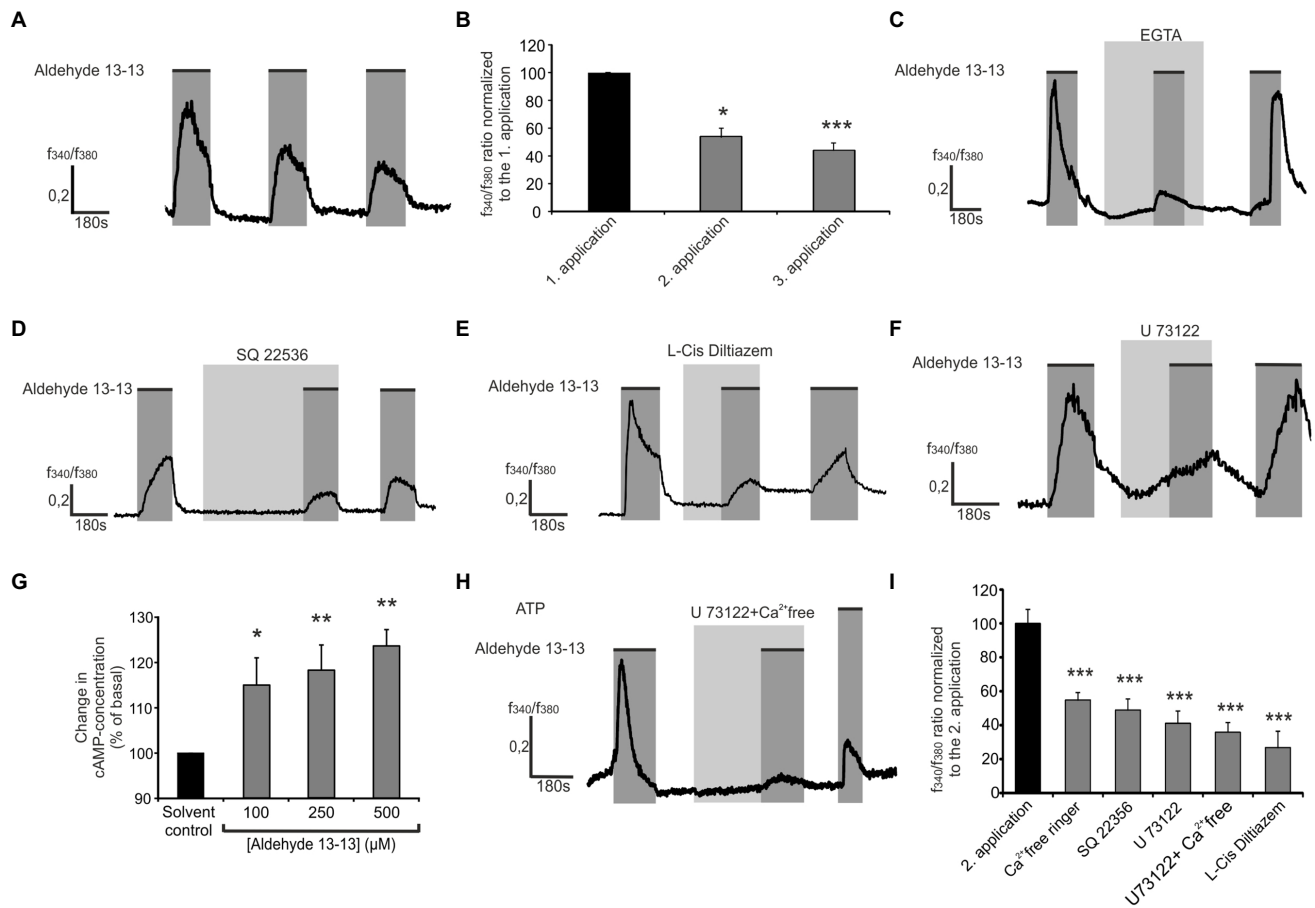
Involvement of the classical olfactory signaling cascade in the aldehyde 13-13 induced increase of intracellular Ca^2+^ in Nthy-ori 3-1 cells. **(A)** Cells were stimulated three times for 3 min with aldehyde 13-13 (500 μM; marked dark gray). **(B)** Quantification of the repetitive applications of aldehyde 13-13 (*n* = 8). **(C)** Preincubation with Ca^2+^ free ringer’s solution containing 10 μM EGTA (marked by a light gray box) reduces the aldehyde 13-13 (500 μM) induced response. Pretreatment with SQ 22536 (10 μM; **D**), L-Cis Diltiazem (100 μM; **E**) or U73122 (5 μM; **F**) reduced the aldehyde 13-13 (500 μM)-induced Ca^2+^ signals in Nthy-ori 3-1. **(G)** Aldehyde 13-13 increases the intracellular cAMP concentration in a dose-dependent manner in Nthy-ori 3-1 cells. **(H)** Exemplary trace of aldehyde 13-13 induced response in Nthy-ori 3-1 cells preincubated with the inhibitor U73122 in Ca^2+^ free ringer solution. **(I)** Quantification of aldehyde 13-13-induced Ca^2+^ signals in blocker experiments relative to control measurements (the average of the second response to aldehyde 13-13 without an inhibitor). Data significance was calculated using One-way ANOVA with Dunnett’s *Post Hoc* test or Friedman Test with “Two-stage step-up method of Benjamini, Krieger, and Yekutieli” referring to the aldehyde 13-13 induced Ca^2+^ signal in control measurements (*n* = 3–12). The mean values are all shown with the corresponding SEM, ^*^p ≤ 0.05, ^**^p ≤ 0.01, and ^***^p ≤ 0.001.

Subsequently, the signal pathway of the aldehyde 13-13 mediated Ca^2+^ response in Nthy ori 3-1 cells was investigated using specific pharmacological inhibitors against signaling molecules to investigate their involvement in the OR2H2 mediated an influx of extracellular calcium. If a signaling molecule is involved in the signaling cascade, the increase in intracellular calcium concentration is reduced by the inhibitor. The amplitudes were compared with those without inhibitor. A repetitive application of the odorant led to desensitization and reduced the occurring amplitudes (Figures 4A,B). For the following experiments, the second amplitude served as control. First, the amplitude of the Ca^2+^ response induced by aldehyde 13-13 (500 μM) was measured under normal conditions in ringer’s solution followed by measurement under Ca^2+^ free conditions, which resulted in a significantly reduced effect (Figure 4C). Then, the involvements of compartments of the classical olfactory signaling cascade in the Ca^2+^ increase were analyzed. For this purpose, the adenylate cyclase (AC) specific inhibitor SQ 22536 (20 μM) was preincubated for 10 min resulting in a significant reduction of the aldehyde 13-13 induced Ca^2+^ amplitude (Figure 4D). Stimulation of OR2H2 with aldehyde 13-13 showed a dose-dependent increase in second messenger cAMP, confirming the involvement of AC in the OR-induced rise in intracellular calcium concentration (Figure 4G). Moreover, the 3-min preincubation with the cyclic nucleotide–gated (CNG) channel-specific inhibitor L-cis-Dilitiazem (100 μM) resulted in a significant reduction of the aldehyde 13-13 mediated response (Figure 4E). A complete blocking was not achieved. Therefore, the additional involvement of another signaling pathway was investigated. The specific phospholipase C (PLC) blocker U73122 (2µM) was preincubated for 5 min and then the odorant was applied with a concentration of 500 μM. The corresponding amplitude was significantly reduced compared to the control amplitude (Figures 4F,I). Finally, U73122 was used under Ca^2+^ free conditions resulting in a significant reduction of the aldehyde 13-13 induced response (Figures 4H,I).

### Aldehyde 13-13 and Nerol Influence Migration, Invasion, and Proliferation of Thyroid Cells

**Figure 5:**
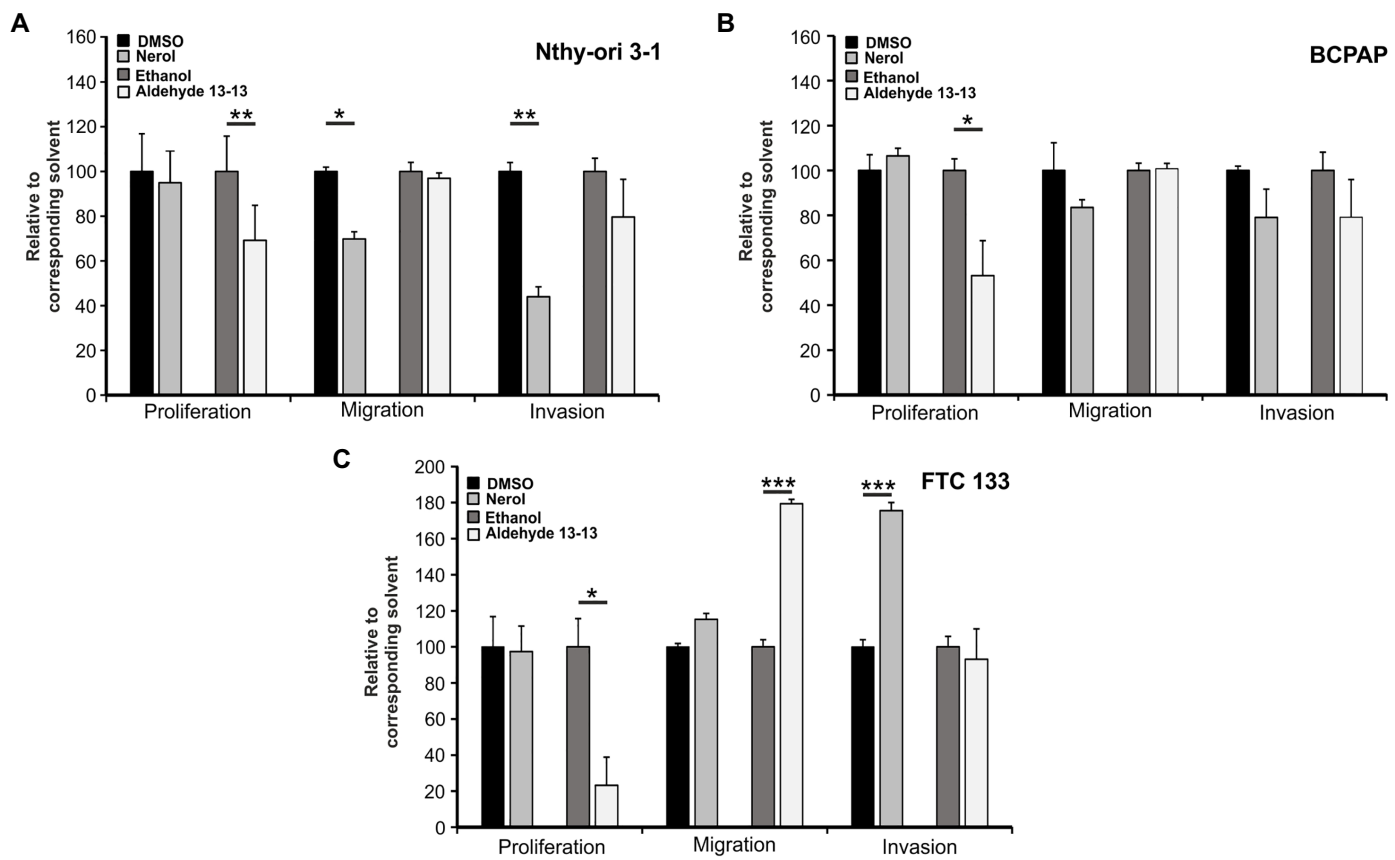
Nerol and aldehyde 13-13 influence migration, invasion, and proliferation of Nthy-ori 3-1 **(A)**, BCPAP **(B)**, and FTC 133 **(C)** cells. Transwell proliferation of the three cell lines treated with nerol or aldehyde 13-13 (500 μM; *n* = 5 in triplicates). In the transwell migration assay, the cells were stimulated with nerol or aldehyde 13-13 with a concentration of 250 μM (*n* = 3–4 in triplicates). In the transwell invasion assay, the Nthy-ori 3-1, BCPAP, and FTC 133 cells were treated with either nerol or aldehyde 13-13 (250 µM; *n* = 4 in triplicates). The percentage of cell migration, invasion, or proliferation of cells/well normalized to the respective solvent control is shown. For all results, the mean values are shown with the corresponding SEM. Data significance was calculated by comparing solvent controls with odorant-treated cells using student’s paired *t*-test. The comparison was made only within cell lines. ^*^p ≤ 0.05, and ^**^p ≤ 0.01, and ^***^p ≤ 0.001.

The functional relevance of the ligands aldehyde 13-13 (OR2H2) and nerol (OR2W3) on human thyroid cells was subsequently investigated *in vitro*. In the transwell migration and invasion assays, nerol significantly reduced the cell migration and invasion of Nthy-ori 3-1 cells compared to the solvent control. Besides, nerol-treated FTC 133 cells were more invasive than the control without affecting the cell migration potential. Furthermore, FTC 133 cells treated with aldehyde 13-13 showed a significantly higher cell migration, whereas aldehyde 13-13 significantly reduced the cell invasion of BCPAP cells (Figures 5B,C).

Moreover, a lower percentage of cells incorporating BrdU is found in line with the reduction of cell proliferation in Nthy-ori 3-1, FTC 133, and BCPAP cells post aldehyde 13-13 application (Figure 5).

## Discussion

Numerous studies in recent years have shown that the expression of ORs is not restricted to the nasal epithelium alone, but occurs almost ubiquitously in the entire body (Zhang et al., 2007; Flegel et al., 2013; Weber et al., 2018). The physiological role of extra nasal ORs has been determined for only a low number of receptors and tissues. In current studies, odorants mediated effects on tumor progression had opened the view on ORs as potential prognostic biomarkers and therapeutic targets (Kim et al., 2015; Maßberg et al., 2016; Weber et al., 2017).

No functional characterization of ORs in human thyroid tissues has been performed so far. The detection of OR2H2 and OR2W3 in the thyroid cell lines Nthy-ori 3-1, FTC 133, and BCPAP, as well as in the corresponding tissues, was performed on the mRNA level by RT-PCR. Multiple works showed an increased expression of ORs in tumor tissues compared to benign (Weber et al., 2018). In contrast, our work showed a reduced expression of OR2W3 in both follicular and papillary thyroid carcinoma tissues when comparing to healthy surrounding tissues. The expression of *OR2H2* was only reduced in follicular thyroid carcinoma tissue. These downregulations of *OR2W3* and *OR2H2* in cancer tissue were in line with the OR expression levels in the associated cell lines. A possible cause for the reduced expression could be a limiting effect of the receptors, as already described for different ORs (Hart et al., 2003; Weber et al., 2018).

The mRNA expression differences could not be confirmed at the protein level. Post transcriptional changes could be an explanation for the observed effects. Here, a variety of possible effects such as turnover rates or 3ꞌ untranslated region (UTR) lengths can impair or promote translation, and thus, result in equal protein expression in the different cell lines despite different mRNA expression (Vogel and Marcotte, 2012). However, the observation must be treated with caution since no quantitative analysis of protein expression was possible. When comparing the receptor protein localization between the cell lines using immunocytochemical staining, no difference was observable. OR2H2 was seen in the nucleus and at the cytoplasmic membrane of the thyroid cell lines, whereas OR2W3 seems to be expressed preferentially in the cytosol and the plasma membrane.

Nuclear expression of GPCRs has also been observed in several tissues (Cavanagh and Colley, 1989; Vaniotis et al., 2011). In addition, OR10AD1 in human retinal cells also shows a predominantly nuclear expression (Jovancevic et al., 2017c). It is likely that the receptors can be transduced to both the membrane and the nucleus by trafficking, as has been described for other G proteins and signaling pathway components (Boivin et al., 2008).

The activating ligand of OR2H2 and OR2W3 has already been identified (Busse et al., 2014; Kalbe et al., 2018). The stimulation of human myoblasts with aldehyde 13-13 increased the intracellular Ca^2+^ concentration and decreased cell differentiation (Kalbe et al., 2018). OR2W3 was detected in sperm and retina but NGS data revealed that it is most strongly expressed in the thyroid gland. However, its functional role has not yet been clarified (Flegel et al., 2015; Jovancevic et al., 2017b). In our study, aldehyde 13-13 and nerol stimulation led to an increase of intracellular Ca^2+^ levels in all thyroid cell lines. In Nthy-ori 3-1 cells, the intracellular Ca^2+^ concentration increased depending on the aldehyde 13-13 concentration until saturation was reached at 1 mM. By knockout of *OR2H2*, its involvement in the increase of intracellular Ca^2+^ concentration induced by aldehyde 13-13 could be confirmed. A weak aldehyde 13-13-induced response was still detectable after knockout of *OR2H2* probably due to OR2H2 protein that was synthesized before transfection and remained in the membrane (Feher, 2017).

To identify the signaling cascade activated by OR2H2 specific inhibitors against pathway components were used. It has been readily shown that a knockout of central pathway molecules leads to reduced viability or morphology (Yan et al., 2012). The inhibitors used were incubated on the cells for a maximum of 20 min to avoid these potentially toxic effects. In addition, the signaling pathways were blocked at multiple targets by different inhibitors. If non-specific effects occur, there is almost no chance that they will also occur with all other inhibitors of this pathway.

Coapplying aldehyde 13-13 and various inhibitors of pathway molecules frequently activated by ORs, it was possible to identify adenylate cyclase III (AC III) as a main actor in the olfactory signaling pathway. The AC III converts ATP to cAMP, which binds to the cyclic nucleotide-gated (CNG) ion channel resulting in a Ca^2+^ influx. This pathway is also induced by ORs in many non-olfactory tissues (Scholz et al., 2016; Tsai et al., 2017; Maßberg and Hatt, 2018). Through the specific blocking of relevant classical signaling molecules, such as AC III, through SQ22536 and the non-selective inhibition of CNG channels for which the subunit CNGA4 and CNGB1 are expressed in thyroid cells, through L-cis Diltiazem, a significant reduction of the Ca^2+^ concentration increase could be observed (Wise, 1996; Wilkinson et al., 2011; Flegel et al., 2013). The results indicate that the canonical signaling pathway is involved in OR2H2 mediated increase in intracellular calcium concentration (Streb et al., 1983; Berridge and Irvine, 1984). This signaling pathway is homogeneous to that which enables odor perception in the nasal epithelium (Pace et al., 1985; Kaupp et al., 1989; Reed, 1992). The reductions were significant but incomplete, which could be explained by the involvement of another signaling pathway. Dual signaling downstream of an OR activation was previously described in olfactory sensory neurons and melanocytes (Breer et al., 1990; Ronnett et al., 1993; Gelis et al., 2016). Phospholipase C (PLC) represents a further key mediator in the OR-induced signaling (Braun et al., 2007; Weber et al., 2017). The involvement of PLC was proved using the specific inhibitor U73122 (Gelis et al., 2016; Jin et al., 2018). The aldehyde 13-13 induced Ca^2+^ response was significantly reduced by application with the blocker. The stimulation with the U73122 under Ca^2+^ free conditions, and therefore, the inhibition of both involved pathways leads to a significant inhibition of the aldehyde 13-13 induced Ca^2+^ response. The inhibition was incomplete which can be explained by the fact that U73122 was used in favor of cell vitality with a concentration of 2 μM, although the IC_50_ value is between 3 and 4 μM (Shears, 1989; Khan et al., 2015). The application of nerol also induced a rise in intracellular cAMP and the intracellular Ca^2+^ level. If a signaling pathway similar to that of aldehyde 13-13 is activated has to be determined in further studies.

To investigate the physiologic relevance of ORs in cells of different tumor types, dysregulated processes such as proliferation, invasion, and migration were analyzed. These processes are known to be regulated by cAMP or IP_3_ signaling (Stork and Schmitt, 2002; Shawl et al., 2009; Cheng et al., 2018), because the activation of the OR usually leads to the activation of exactly these pathways, ORs seemed like suitable therapeutic targets (Maßberg and Hatt, 2018). It was already shown in various cell types, e.g., prostate cancer cells, melanocytes, and airway smooth muscle cells, that the activation of ORs leads to a reduction of proliferation (Neuhaus et al., 2009; Aisenberg et al., 2016; Wojcik et al., 2018). Stimulation with aldehyde 13-13 also resulted in reduced proliferation in all analyzed thyroid cell lines. Additionally, stimulation of BCPAP cells with aldehyde 13-13 resulted in a decreased invasion, while Nthy-ori 3-1 and FTC 133 were not affected. The cell migration was only increased in FTC 133 after aldehyde 13-13 stimulation. The results shown support the initial theory that ORs may be downregulated due to a limiting effect. Taking migration as an example, Nthy-ori 3-1 cells showed the highest OR2W3 expression and stimulation of these with nerol resulted in a decrease. A similar pattern is seen for OR2H2, which is weaker expressed in FTC 133 than in Nthy-ori 3-1 and BCPAP and after stimulation only in the FTC 133 cell line causes increased migration. The impact of OR stimulation on cell migration and invasion was additionally previously described by Sanz et al. (2014) and Kim et al. (2015).

As observed in this study, physiological consequences vary between cell types after stimulation with the same odorants. This observation was also made in the epithelium of hair follicles and chronic myelogenous leukemia cells (Manteniotis et al., 2016a; Chéret et al., 2018).

In comparison, stimulation with nerol led to increased migration and invasion of Nthy-ori 3-1 cells. Moreover, nerol stimulation did not affect cell proliferation at all. A comparable OR mediated effect has already been observed in other tumor types such as prostate cancer, where the increased invasion was due to PI_3_K activation (Sanz et al., 2014; Cao et al., 2015).

Consequently, nerol and aldehyde 13-13 stimulation leads to different physiological consequences in the same cell type. This observation that different odorants have a different effect on the same cell type was also described in human airway smooth muscle (Kalbe et al., 2016).

Based on this, we hypothesize that either different signaling pathways are activated by aldehyde 13-13 and nerol or that the signaling pathway components are differentially expressed. It was previously described that OR can activate different signaling pathways, even in the same cell (Scholz et al., 2016). For a more precise explanation, the single steps between activation of the receptor and the induced physiological effect should be analyzed in detail in further studies.

Although aldehyde 13-13 and nerol are no endogenous substances, they are able to activate OR2H2 or OR2W3 expressed in thyroid cells. In these cells, the ORs mediate various effects such as calcium balance, migration, invasion, or even the synthesis of second messengers. Based on the results shown in this work, future molecular modeling studies could address the search for endogenous ligands and thus identify a new regulatory mechanism.

In sum, we demonstrate that ORs such as OR2H2 and OR2W3 are not only expressed in healthy human thyroid tissue but also human thyroid cancer cells and respond to their ligands *via* a combination of cAMP and IP_3_ dependent signaling. *In vitro* characterization confirmed that ligand OR2H2 and OR2W3 interaction has a functional impact on cancer biology, increasing the invasive potential of follicular thyroid cancer cells. These studies show the multifactorial effects of GPCRs on healthy and diseased thyroid tissue’s biological behavior.

## Data Availability Statement

The raw data supporting the conclusions of this article will be made available by the authors, without undue reservation.

## Ethics Statement

The studies involving human participants were reviewed and approved by Clinical Ethics Committee of the University Hospital Essen. The patients/participants provided their written informed consent to participate in this study.

## Author Contributions

DW, DZ, ST, and JH performed the research. DW, NJ, DZ, DF, and HH designed the research study. DW and NJ wrote the original draft and analyzed the data. DZ, DF, and HH reviewed and edited the draft. HH and DF contributed to the project administration. All authors contributed to the article and approved the submitted version.

## Conflict of Interest

The authors declare that the research was conducted in the absence of any commercial or financial relationships that could be construed as a potential conflict of interest.

## Publisher’s Note

All claims expressed in this article are solely those of the authors and do not necessarily represent those of their affiliated organizations, or those of the publisher, the editors and the reviewers. Any product that may be evaluated in this article, or claim that may be made by its manufacturer, is not guaranteed or endorsed by the publisher.
