# Supplementary material for: Functional Characterization of Olfactory Receptors in the Thyroid Gland
Source: Front Physiol. 2021 Jul 27;12:676907. doi: 10.3389/fphys.2021.676907 (PMC8353271; doi:10.3389/fphys.2021.676907)
Supplement: Supplementary file 1 [file Data_Sheet_1.docx]

Supplementary Material

# Supplementary Figures:


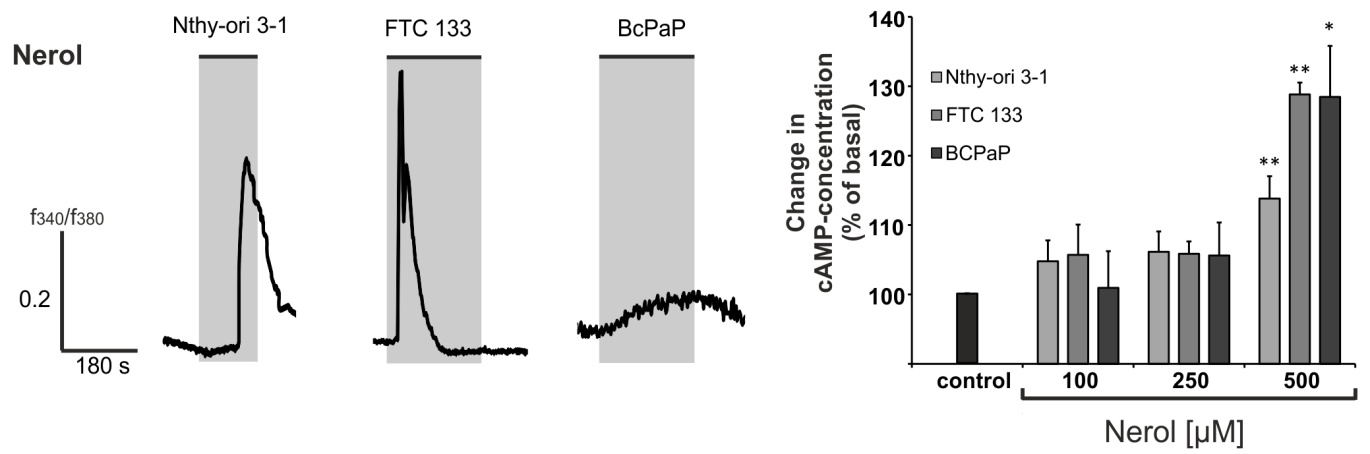


**Figure S1: Nerol induces an increase of intracellular Ca^2+^** **via cAMP by the stimulation of OR2W3.** (A) Exemplary traces of Ca^2+^ responses induced by stimulation with 500 µM nerol in the thyroid cell lines. (B) Intracellular cAMP concentration increases in thyroid cells after stimulation with nerol. The cells were stimulated for 30 min with different concentrations of nerol. Afterwards, the intracellular cAMP concentration was compared to solvent control. The mean values are shown with the corresponding standard error of the mean, n=5 in triplicates, student paired t-test, *=p≤0.05 and **=p≤0.01.


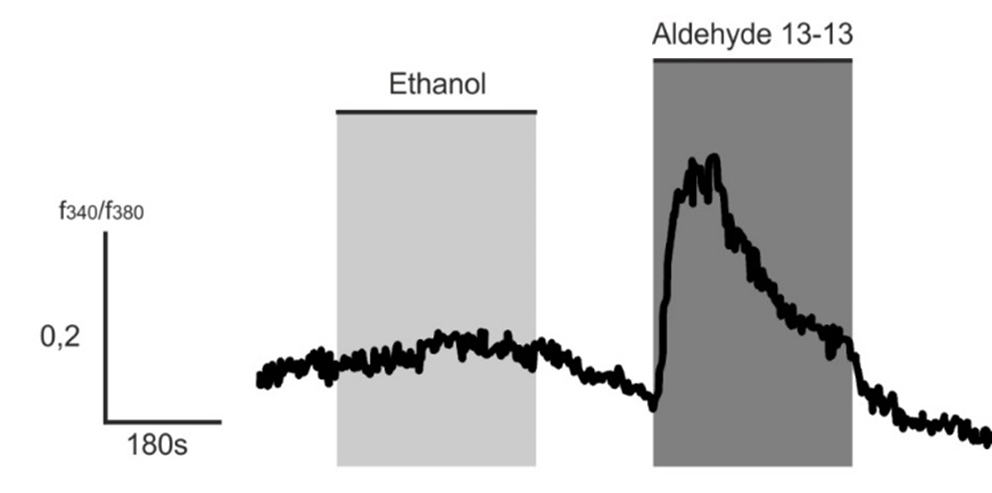


**Figure S2: Ethanol has no effect on intracellular calcium level of Nthy-ori 3-1 cells.** 0.1% Ethanol was applied, which corresponds to the maximum concentration in the odorant’s applications. The aldehyde 13-13 concentration was 500 µM.
